# Single nucleotide variant discovery of highly inbred Leghorn and Fayoumi chicken breeds using pooled whole genome resequencing data reveals insights into phenotype differences

**DOI:** 10.1186/s12864-016-3147-7

**Published:** 2016-10-19

**Authors:** D. S. Fleming, J. E. Koltes, E. R. Fritz-Waters, M. F. Rothschild, C. J. Schmidt, C. M. Ashwell, M. E. Persia, J. M. Reecy, S. J. Lamont

**Affiliations:** 1 Iowa State University, Ames, IA USA; 2 University of Delaware, Newark, DE USA; 3 North Carolina State University, Raleigh, NC USA; 4 Virginia Polytechnic and State University, Blacksburg, VA USA; 5 Department of Animal Science, University of Arkansas, Fayetteville, AR 72701 USA

**Keywords:** Resequencing, Single nucleotide variant, Genomic diversity

## Abstract

**Background:**

Analyses of sequence variants of two distinct and highly inbred chicken lines allowed characterization of genomic variation that may be associated with phenotypic differences between breeds. These lines were the Leghorn, the major contributing breed to commercial white-egg production lines, and the Fayoumi, representative of an outbred indigenous and robust breed. Unique within- and between-line genetic diversity was used to define the genetic differences of the two breeds through the use of variant discovery and functional annotation.

**Results:**

Downstream fixation test (*F*_*ST*_) analysis and subsequent gene ontology (GO) enrichment analysis elucidated major differences between the two lines. The genes with high *F*_*ST*_ values for both breeds were used to identify enriched gene ontology terms. Over-enriched GO annotations were uncovered for functions indicative of breed-related traits of pathogen resistance and reproductive ability for Fayoumi and Leghorn, respectively.

**Conclusions:**

Variant analysis elucidated GO functions indicative of breed-predominant phenotypes related to genomic variation in the lines, showing a possible link between the genetic variants and breed traits.

**Electronic supplementary material:**

The online version of this article (doi:10.1186/s12864-016-3147-7) contains supplementary material, which is available to authorized users.

## Background

With the availability of new and more powerful next generation sequencing technologies, massive amounts of molecular data can be generated from individual or pooled genomic DNA samples. Discovery and characterization of variants within and among individuals allows definition of the genetic dissimilarities that may underlie phenotypic variation. Genome resequencing data from within-population pooled samples can be effectively used to characterize genetic variation within and between populations, and to accurately estimate allele frequencies [1]. There are multiple other advantages to pooling samples to generate data, including cost-effectiveness and accuracy [1]. Pooled whole genome resequencing has been used to conduct association studies with phenotypes of interest and to identify signatures of selection [2, 3]. Genome resequencing data can also be used to evaluate evolutionary changes and define the phylogenetic relationships between divergent members of a species [4, 5].

Since domestication, the selection for specific traits and directed evolution in livestock has led to the creation of different breeds or a “Domestication Phenotype” within a species [5]. The chicken breeds used to establish the experimental lines analyzed in the present study represent diverse selection histories. The Fayoumi and Leghorn breeds differ in many morphological features and phenotypes, and commercial relevance. Under the “Domestic Phenotype” concept, the Leghorn represents a specialized breed driven by artificial selection for improved egg production traits. The Leghorn breed is native to Italy and was selected for reproduction traits as early as the Roman Empire [6]. The Fayoumi breed is native to Egypt, where it was prized for its robustness in a harsh environment. The breed was imported to the USA because of it’s reported resistance to viral disease [7, 8]. The lines used in this study differ in their response to Marek’s disease (MD) virus, in that Fayoumis had significantly fewer tumors and clinical signs of MD [9, 10] than Leghorns. In addition, Fayoumi chickens had lower mortality and lesion scores than Leghorns after infection with *Eimeria tenella*, a parasite that causes coccidosis [11]. For the purpose of the current study, the lines were characterized by the phenotype designation of “immune function based” traits for Fayoumi and “reproduction based” traits for Leghorn [12]. These traits are relative descriptors of the major phenotypes that contrast between the two lines, and reflect the original breeds’ known history of natural and artificial selection. These “breed phenotypes” serve as the anchor to inform how we interpret the results from variants analyzed in the Fayoumi and Leghorn populations. These analyses focus on fixed unique, line-specific alleles that are different than the Red Jungle Fowl reference sequence. The objective of our study was to analyze variants that define the genomic architecture and line-specific differences of highly inbred Leghorn and Fayoumi chicken lines.

## Results and discussion

### Variant discovery

The Fayoumi and Leghorn pooled sequence data were each compared against the Red Jungle Fowl (RJF) reference genome (Galgal4) to identify single nucleotide variants (SNV) and insertion/deletions (indels) present in these populations (Table 1). Analysis versus the RJF revealed a total of about 4 million variants each for the Fayoumi and Leghorn lines. There were 1,238,884 Fayoumi and 1,318,012 Leghorn variants that were present in dbSNP, whereas 3,223,583 Fayoumi and 3,287,720 Leghorn variants were previously uncharacterized (not previously submitted to dbSNP). The overall genome homozygosity percent for both inbred lines was approximately 99.95 %. These extremely low levels of within-line variation support our expectation that most alleles would be fixed, given the stringent and long inbreeding of these two populations.

**Table 1:** Variants discovered by breed type

	Fayoumi	Leghorn	^a^Fayoumi vs. Leghornized reference
Depth of coverage	~24×	~22×	~24×
Assembly coverage	84.5	93.7	84.5
Total variants	4,462,467	4,605,732	3,792,327
Previously uncharacterized variants	3,223,583	3,287,720	3,791,430
Homozygosity	99.9482 %	99.9736 %	99.9330 %
^b^Ts/Tv (All Variants)	2.371	2.365	2.286
Change rate	1 change every 235 bases	1 change every 227 bases	1 change every 276 bases

Total reads computed using GATK DepthofCoverage. Assembly coverage and Sequence coverage calculated using Samtools and GATK DepthofCoverage. All other data calculated using SnpEff. All data is pre-filter. ^a^Fayoumi vs. Leghorn only compared using SNV data; indels were excluded along with Chromosomes Z and W. ^b^Ts/Tv is the ratio of transitions/transversions within each population

Table 2 shows the variants for each line by variant type (homozygous and heterozygous) and bird genotype (Leghorn and Fayoumi). Compared by type, the total number of variants within each population is very similar. However, Fayoumis had more than twice the number of heterozygous variants than did Leghorns. The effects of the variant on the populations are shown in Table 3. For both breeds, most of the variant’s effects were either intronic or intergenic (Table 3). Over 70 % of variants are silent or nonsense mutations across the genome of the Fayoumi and Leghorn populations, and both populations had similar missense/silent ratios (Table 4). For the effects by type or functional consequence, there were significantly (*P < 0.01*) more variants annotated as downstream, upstream, start_gained, and frame_shifts in Leghorns than Fayoumis.

**Table 2:** Comparison of variant changes for each line

Type	Total	Homozygous	Heterozygous
Fayoumi vs. RJF^b^			
SNV	4,146,394	3,638,803	507,591
INS^c^	180,752	158,256	22,496
DEL^d^	135,321	125,514	9,807
TOTAL	4,462,467	3,922,573	539,894
Leghorn vs. RJF			
SNV	4,271,399	4,010,609	260,790
INS	189,494	177,474	12,020
DEL	144,839	142,181	2,658
TOTAL	4,605,732	4,330,264	275,468
Fayoumi vs. Leghorn: reference^a^			
SNV	3,792,327	3,094,177	698,150
TOTAL	3,792,327	3,094,177	698,150

Fayoumi and Leghorn vs. RJF and Fayoumi vs. the Leghornized reference genome. ^a^Fayoumi vs. the Leghornized reference genome analysis done on SNVs only

^b^ *RJF* Red Jungle Fowl, ^c^ *INS* insertion variants, ^d^ *DEL* deletion variants

**Table 3:** Variant annotations and counts by effect type for each line

Effect Type	Fayoumi	Leghorn	Chi-Square Statistic
Codon_Change_Plus_Codon_Deletion	16	24	
Codon_Change_Plus_Codon_Insertion	23	34	
Codon_Deletion	40	53	
Codon_Insertion	46	63	
Downstream	401,163	440,064	*P* < 0.0001
Exon	418	473	
Frame_Shift	384	504	*P* < 0.0001
Intergenic	2,344,623	2,430,279	*P* < 0.0001
Intron	2,205,047	2,264,238	*P* < 0.0001
Non_Synonymous_Coding	14,335	16,924	*P* < 0.0001
Non_Synonymous_Start	5	2	
Splice_Site_Acceptor	229	236	
Splice_Site_Donor	196	251	*P* < 0.01
Start_Gained	1,015	1,185	*P* < 0.01
Start_Lost	35	41	
Stop_Gained	107	121	
Stop_Lost	16	18	
Synonymous_Coding	37,502	41,860	*P* < 0.0001
Synonymous_Stop	9	12	
Upstream	397,941	438,052	*P* < 0.0001
Utr_3_Prime	48,430	52,433	*P* < 0.0001
Utr_5_Prime	6,445	7,802	*P* < 0.0001

Table shows variant annotations and counts for Fayoumi and Leghorn populations vs. RJF by effect type. The “effect type” is the sequence ontology meaning for example that the variant hits an intron or causes a frameshift. A Pearson’s chi-square goodness-of-fit test was used for comparison (*P* < 0.01)

**Table 4:** Variant totals by mutation type

	Mutation	Count	Percent
Fayoumi	Missense	14,389	27.6 %
	Nonsense	106	0.2 %
	Silent	37,512	72.1 %
Leghorn	Missense	16,982	28.7 %
	Nonsense	118	0.2 %
	Silent	41,873	71.0 %

The Missense/Silent ratio: 0.3836 for Fayoumi and 0.4056 for Leghorn populations respectively

There was no difference in the number of variants with exon effects, but there was a difference in those in intergenic regions (Table 3), with more in Leghorn than in Fayoumi. There was also a significant (*P < 0.01*) difference in the number of variants that had an effect either upstream or downstream of their location (Table 3), with more in Leghorn than in Fayoumi.

### SNV validation

One hundred SNVs were selected for wet-lab validation to ascertain the ability of the bioinformatics methods and the pooled-line resequencing data to correctly identify point mutations and provide allele frequency information. Of these 100 SNVs, 37 were specific to the Fayoumi population, 36 were specific to Leghorn, and 27 were in common between the two populations. Sixty-one assays were usable for validation (25 SNVs were clustered too closely and caused primer interference, and another 14 assays failed for technical reasons related to the PCR plates). Of the 61 usable assays for validation of the presence of an SNV, over half (38) showed evidence of duplication based on analysis of the KBiosciences Kompetitive Allele Specific PCR genotyping system (KASP) KlusterKaller software output [13]. Duplication was also indicated by the number of reads that covered the SNVs compared to the mean depth of coverage, with both populations having spikes in these regions in the mean number of reads mapped to a SNV position (Table 5). Quantifying the allele frequencies of the variants showing duplication may have been complicated by the young age of the duplications, because more recent duplications would still be similar to each other. The variants in duplications tended to have with-in population allele frequencies that were close to 50/50 and would often appear as all heterozygous calls in both the study and control populations. The primers designed for the variant and reference base may have amplified different binding sites, preferentially revealing segmental duplications or areas with high sequence homology. This phenomenon is supported by the presence of clusters that fall between homozygous and heterozygous clusters suggesting a 3:1 allele ratio (i.e. G/G and A/G). Additionally, sequencing and mapping errors can reduce accuracy of variant calling. It is improbable that the SNVs validated as heterozygous-only calls that also showed evidence of duplications were not actually duplications, but rather every bird was truly heterozygous for that allele. The validation results show more segregation within the inbred Fayoumis, which agrees with the discovery software but may be an artifact of number of assays that passed validation. The results also suggest that the software programs used for discovery and annotation can be used to discover valid SNVs, but will also identify duplications within the populations. The results from the validation were used to inform the use of a second level of strict filtering parameters applied to the variant discovery data. These parameters were used to support the results from the gene ontologies uncovered using the exploratory filtered data.

**Table 5:** Classification of SNVs used for validation

Class	Fayoumi SNVs	Leghorn SNVs	Common SNVs	Total	Classification description
A	5	0	0	5	Segregating in population only
B	14	0	0	14	Fayoumi and Leghorn different (one segregating, one not segregating), and segregating in controls
C	2	0	0	2	Fayoumi, Leghorn, and controls mix of homozygous for reference or alternate allele, but no heterozygotes
D	4	10	0	14	Failed
E	11	24	2	37	Evidence of duplication
F	1	2	0	3	Only Homozygous (Fayoumi and Leghorn homozygous for different alleles, segregating in controls)
Total	37	36	2	75	
					Pass rate = 81.3 % (75–14)/75

Table shows the results of wet-lab validation of 100 uncharacterized SNVs. The data from validation was used to inform the additional filtering steps used in downstream analysis (strict-filter) of the within-line variation

### Within-line variation

The lines used in this study have not undergone genetic selection since their establishment in 1954, with the exception of selection for adequate reproduction in both lines. The fixed or segregating variation should represent a combination of natural selection, genetic drift, and mutation in the Fayoumi and Leghorn lines. To categorize the potential impact of variations, the annotation program groups the functional impacts of variants as high, moderate, low and modifier. These impact categories were analyzed for fixed, unique (line-specific) variants from either Fayoumi or Leghorn using gene ontology enrichment analysis. Fixed unique variants for each line are assumed to represent either the alleles that were selected during domestication, breed selection, or those fixed during the over 60-year process of inbreeding. There were no statistically over-represented GO terms in the analysis of the high and low impact variants. However, variants that were classified as moderate impact were statistically associated with GO terms (Table 6). Some of these variants are shared but with differences between breeds as shown in the *F*_*ST*_ analysis.

**Table 6:** Overrepresented gene ontology terms for moderate impact^b^, line-specific variants in Fayoumi and Leghorn lines

Fayoumi GO Term	Count	*P*-value^a^
*FN3*	22	1.10E-03
Ribonucleotide binding	154	1.70E-03
Purine ribonucleotide binding	154	1.70E-03
Fibronectin, type III	22	8.10E-03
*PTPc*	8	1.70E-02
Nucleotide binding	177	2.10E-02
Protein kinase activity	61	2.60E-02
Leghorn GO Term	Count	*P*-value^a^
ECM-receptor interaction	25	9.60E-05
Extracellular matrix	41	1.90E-04
Metal ion binding	270	2.30E-03
Proteinaceous extracellular matrix	37	1.30E-03
Extracellular region	94	2.30E-03
Cell division and chromosome partitioning	26	2.60E-02
Calcium ion binding	77	3.90E-02
Aminophospholipid transporter activity	7	4.30E-02
Phospholipid-translocating atpase activity	7	4.30E-02

Over-represented GO terms for moderate impact variants (fixed/segregating) and unique for the inbred Fayoumi and Leghorn lines. ^a^Benjamini-Hochberg Corrected *p-value* cut-off *α = 0.05.* ^b^Moderate impact variants: non_synonymous_coding, codon_change, codon_insertion, codon_change_plus_codon_insertion, codon_deletion, codon_change_plus_codon_deletion, utr_5_deleted, utr_3_deleted

### Within-line variation: Fayoumi

For Fayoumi, whose breed phenotype was considered to be immune-related, the GO terms fibronectin type III (*FN3*), and tyrosine-protein phosphatase 3 (*PTPc*) were statistically significant (*adjusted p value < 0.05*; Table 7). Fibronectin type III is a multidomain glycoprotein found in connective tissue and binds actin and DNA along with other substances thereby aiding defense against pathologies. Fibronectins enhance wound closure, cell adhesion, and blood coagulation [14]. The ubiquitously expressed domain has also been shown to involved in cytokine signaling and may play a role in the efficiency of the Fayoumi innate immune system [15, 16].

**Table 7:** Gene ontology terms from DAVID for variant regions with greatest difference (*F*_*ST*_ *= 1*)

GO Terms	Count	*P*-value
Nucleoside binding	630	6.90E-13
Purine nucleoside binding	626	4.30E-13
Adenyl nucleotide binding	622	3.50E-13
Nucleotide binding	869	9.10E-13
Purine nucleotide binding	745	2.90E-12
Adenyl ribonucleotide binding	585	1.20E-11
ATP binding	581	1.70E-11
Ribonucleotide binding	708	5.20E-11
Purine ribonucleotide binding	708	5.20E-11
Protein kinase activity	263	1.60E-07
Protein amino acid phosphorylation	273	7.30E-05
Atp-binding	243	1.50E-05
Nucleoside-triphosphatase regulator activity	129	1.80E-05
Gtpase regulator activity	125	3.10E-05
Protein serine/threonine kinase activity	168	3.70E-05
Extracellular ligand-gated ion channel activity	49	4.00E-05
Nucleotide-binding	300	1.00E-04
Phosphorus metabolic process	361	9.20E-04
Phosphate metabolic process	361	9.20E-04
Enzyme activator activity	78	2.90E-04
Nucleotide phosphate-binding region: ATP	103	2.50E-02
Gtpase activator activity	63	1.10E-03
Identical protein binding	112	1.50E-03
Ligand-gated ion channel activity	64	1.50E-03
Ligand-gated channel activity	64	1.50E-03

Functional categories from DAVID representing the genes that had *F*_*ST*_ value’s of 1. GO Terms from DAVID based on *F*_*ST*_ values of 1 for comparison of variant position between populations. Benjamini Corrected *p*-value cut-off *α = 0.05*

The expression of genes related to cell adhesion signaling has been recently shown to play a role in viral immune responses [17]. Fibronectins are reported as being “subject to high selective pressure” [18], which is in agreement with their apparent fixation in the current study. Genes annotated to the GO term *FN3* included the leptin receptor (*LEPR)*, a member of the cytokine receptor superfamily, adipocytokine, and JAK-STAT signaling pathways that promote inflammatory responses to pathogens, as well as angiogenesis and cellular repair processes. Leptin receptor expression can also affect follicle formation in breeder hens [19]. After strict filtering of the variants based on the information from the validation and the exploratory run to collect information on genomic diversity, the variations associated with fibronectin type III still remained statistically significant (*FDR ≤ 0.05*). However *PTPc* disappeared and was replaced with statistically significant GO terms for cytokine-cytokine receptor interactions and natural killer cell (Additional file 1: Table S1.). Over-enriched terms from each analysis point to possible connections between breed-specific variants and their breed phenotypes.

The Prolactin receptor (*PRLR*) was represented in the Fayoumi gene list under the GO term *FN3*. The prolactin receptor has a role in egg production in chickens [20, 21] and is also a member of the cytokine signaling superfamily, giving it some influence on immune functions [22]. The genes *LEPR* and *PRLR* both were present in the statistically significant GO term lists based on the exploratory and strict variant filter lists. Both genes showed a decrease in the number of variants associated with all functional impacts (*LEPR* exploratory filter = 245, strict filter = 95; *PRLR* exploratory filter = 104, strict filter = 65) based on filtering, but still remain associated to the over-enriched *FN3* term.

### Within-line variation: Leghorn

Within-population analysis of the fixed and unique Leghorn variants different from the RJF reference, revealed GO terms related to reproductive phenotypes. Gene set enrichment analysis of the moderate impact variants gave significant results (*adjusted p value < 0.05*) for the GO terms of cell division, calcium ion binding, phospholipid activity and extracellular matrix annotations (Table 6), all processes involved in egg production [23]. It is possible that variants in these gene clusters are tolerated and may represent sets of diversification/improvement genes. Genes of interest within the extracellular matrix cluster include *TIMP*, metallopeptidase inhibitor 2 (*TIMP2*), an inhibitor of metalloproteineases that degrades the extracellular matrix and suppresses endothelial cell proliferation. Highly conserved in multiple species, *TIMP2* plays a role in chicken eggshell production and embryogenesis [24, 25]. Calcium ion binding and metal ion binding (Table 6) are related to reproductive phenotypes for the Leghorn as the genes within these categories are involved in mineral and ion transport in the chicken uterus [25]. Another gene of interest involved in calcium ion binding is the gene epidermal growth factor (*EGF*) which affects multiple pathways including extracellular growth and differentiation, focal adhesion, and MAPK signaling pathways [26]. Along with calcium ion binding, there are also genes in the gene list that function as ion transporters and in the case of the aminophospholipids help form bilayers [24].

There are many variants driving overlapping functions and genes between lines that appear as over-enriched. One such gene is *PRLR,* which is essential for egg production and has impacts on immunological functions. The uniqueness in the number of variants, position, and effects suggests that the two lines evolved different uses for this gene. It is unknown if variants for genes grouped into functional classes such as calcium binding and phospholipid-translocating ATPase activity for Leghorn, or *FN3* for Fayoumi, actually convey an advantage or disadvantage to egg production or disease resistance. Over time, the natural selection for survivability and reproduction may have contributed to differences or similarities in the genetic architecture of reproduction traits, leading to convergent phenotypes.

Strict filtering of the variants for the Leghorn (Additional file 1: Table S1) showed that calcium ion binding and extracellular matrix remained statistically significant but the gene *TIMP2* associated with extracellular matrix is lost but was replaced by the GO term metallopeptidase activity. The strict filtered gene list also picked up information on cadherins, glycerophospholipids, and proteoglycans shown to be involved in eggshell matrix, Ca^2+^ mediated cell-cell adhesion, and egg lipid matrix generation during reproduction in hens [25, 27]. Additionally the genes *TIMP2* and *EGF* showed a decrease in the number of variants associated with all functional impacts, with the gene *TIMP2* disappearing completely from the list (*TIMP2* exploratory filter = 112, strict filter = 73; *EGF* exploratory filter = 440, strict filter = 413; *PRLR* exploratory filter = 167, strict filter = 113) based on filtering.

### Between-line variation

The Fayoumi and Leghorn pooled sequence data were compared against the RJF reference to call possible SNVs and indels present in the populations. Of these variants, 1,238,884 (27.8 %) have been previously deposited in dbSNP at the time of analysis, generating 3,223,583 (72.2 %) previously uncharacterized variants called within the Fayoumi experimental line. Similar results were obtained in the analysis of the Leghorn resequencing data vs. RJF reference assembly. A total of 4,605,732 variants were discovered of which 3,287,720 (71.3 %) were previously uncharacterized. We also examined the number of variants in each population using SnpEff categories of “effects by type” and “effects by region”. A subset of 2,052,537 variants was unique to Fayoumi and 2,196,553 unique to Leghorn after removal of variants common to both groups.

To help characterize the differences and similarities between the inbred populations of Fayoumi and Leghorn chickens, a fixation index (*F*_*ST*_) analysis was conducted using the program PoPoolation2 [28], which employs the Karlsson *F*_*ST*_ method [2]. The *F*_*ST*_ value between the populations was calculated for each gene represented in the variant call file output based on the allele frequencies at every base for each gene. The *F*_*ST*_ analysis generated a list of genes with *F*_*ST*_ values of 1 in which the structure of the two lines showed the most differentiation. Further analysis, using DAVID, of the genes from this analysis indicated that the two lines mainly differed in nucleoside and nucleotide binding, catalytic activity, and ATP usage (*adjusted p < 0.05*). The DAVID (Table 7) output was further processed in REViGO (Table 8) to identify additional unique over-represented GO terms. Gene ontology annotations emerged for population-level differences in the variants that each breed may use to drive various processes. Annotations for immune system processes, response to stimulus, and metabolic processes (*adjusted p value < 0.05*) were over-represented terms that emerged from the GO analysis of the *F*_*ST*_output. The biological processes represented by ontology terms such as immune system process are consistent with the historical breed phenotype for the Fayoumi and may be related to the reported phenotypic differences in pathogen resistance between the breeds [11, 29, 30]. Other over-represented ontology terms identified by the *F*_*ST*_ and GO analyses included: biological adhesion, developmental process, and cellular protein modification process (*adjusted p value < 0.05*). It is possible that these terms may be associated with the Leghorn breed’s historical breed phenotype of selection for egg production [31, 32]. The terms highlighted by the GO analysis are all facets of metabolic processes and indicate that, when each inbred population is characterized by the traits of immune response and reproductive/production ability, the two populations share limited genetic similarity based on these GO annotations. The amount of overlap between the two lines was determined by examination of the variants that were unique or common, and those that were fixed or segregating, for each population (Table 9). The variants “common” to the two lines were based on the same position and allele frequency of the variant in that position.

**Table 8:** Gene ontology terms from REViGO for variant regions with greatest difference (*F*_*ST*_ *= 1*)

Description	Frequency	Uniqueness
Immune system process	0.86 %	0.99
Cellular protein modification process	2.99 %	0.83
Behavior	0.09 %	0.92
Metabolic process	78.07 %	1
Cellular process	70.74 %	1
Cellular component organization	4.20 %	0.95
Sexual reproduction	0.08 %	0.99
Biological adhesion	2.09 %	0.99
Signaling	5.13 %	0.99
Multicellular organismal process	1.33 %	0.99
Developmental process	1.67 %	0.99
Growth	0.14 %	0.99
Locomotion	3.09 %	0.99
Single-organism process	25.74 %	1
Single-multicellular organism process	1.30 %	0.8
Positive regulation of biological process	0.84 %	0.76
Anatomical structure development	1.38 %	0.89
Response to stimulus	10.51 %	0.99
Localization	17.22 %	1
Multi-organism process	4.65 %	0.99

REViGO visualization showing the most unique GO terms represented by the *F*_*ST*_ list of genes for the comparison of the population structures of the Fayoumi and Leghorn. The list reveals terms such as immune system processes and sexual reproduction that represent the traits for which each breed is characterized

**Table 9:** Genomic annotations and count of variants for Fayoumi vs. Leghorn: reference^a^

Type (Fayoumi vs. Leghorn: reference^a^)	Count
DOWNSTREAM	161,833
EXON	300
INTERGENIC	872,171
INTRON	812,038
NONE	2,121,836
NON_SYNONYMOUS_CODING	26,500
NON_SYNONYMOUS_START	16
SPLICE_SITE_ACCEPTOR	602
SPLICE_SITE_DONOR	634
START_GAINED	507
START_LOST	31
STOP_GAINED	1,511
STOP_LOST	53
SYNONYMOUS_CODING	21,093
SYNONYMOUS_START	21
SYNONYMOUS_STOP	27
UPSTREAM	158,646
UTR_3_PRIME	23,377
UTR_5_PRIME	3,428

Counts by region are based on SNVs only. ^a^Alternate reference genome

The shared segregating alleles between Fayoumi and Leghorn indicates that variable changes in these genes may have fewer consequences on gene functions or that variability within these genes is necessary at this position and resists fixation to aid proper genomic integrity in chickens. There are also the shared fixed variants in which the position of the variant is fixed for the same alternative variant in both populations. The fixed variants most likely represent alleles present at the time of domestication [4, 33]. In contrast to the “common” overlapping variants, there are SNVs and indels that are unique to Fayoumi or Leghorn, but share the same genome base pair position. The difference in the called variants and their effects on the gene function in both breeds at similar positions may indicate that these alleles became fixed by positive selection after domestication and can be considered alleles in diversification or improvement genes. The grouping of genes identified by variants in the categories of fixed and unique (i.e., differing in the alternate allele) was used as our model to examine the within line variations of each inbred population of chicken to characterize genetic variation.

### Fayoumi vs. Leghorn: alternate reference

**Fig. 1:**
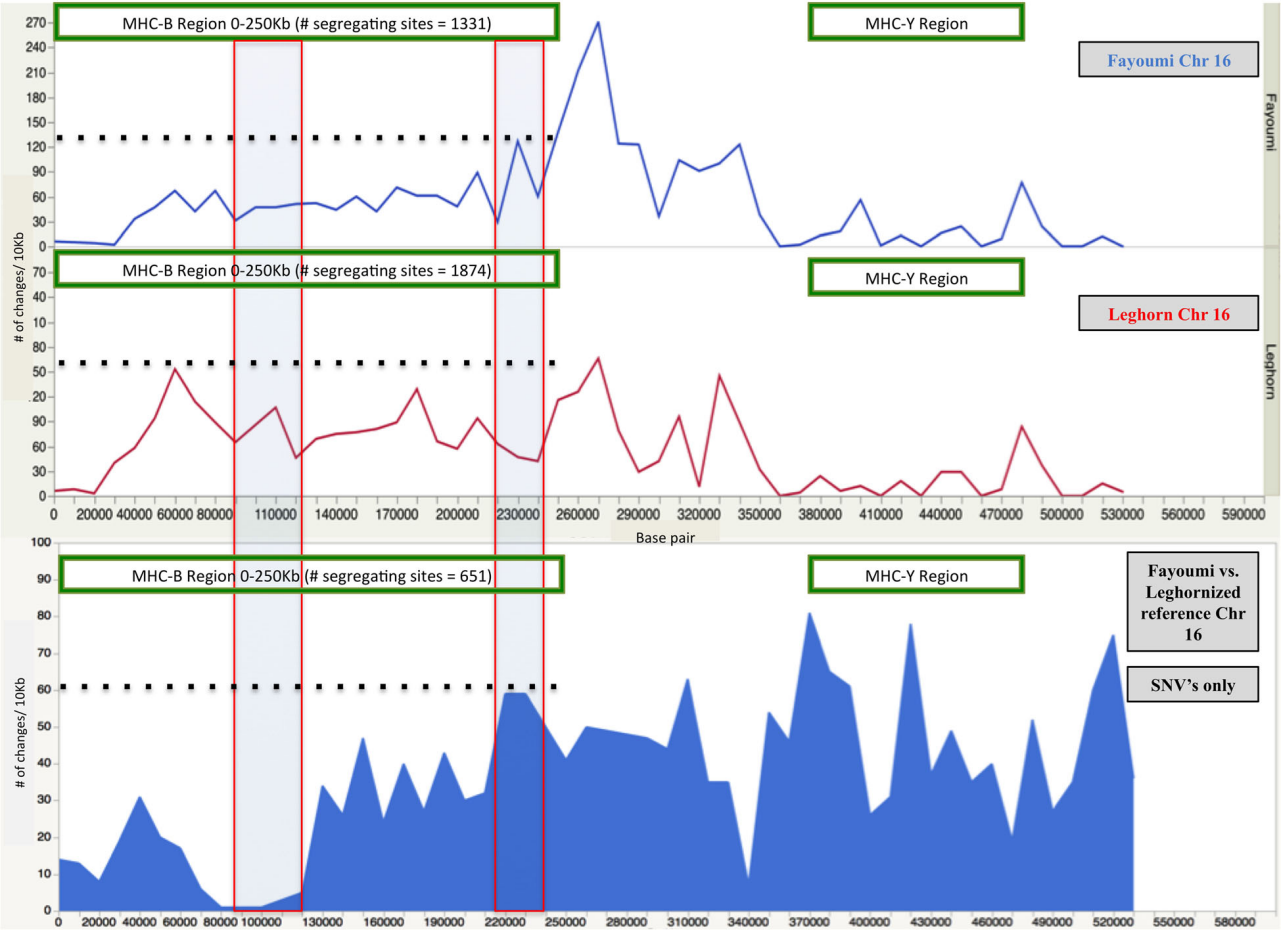
Chromosome 16 variants/10 kilobase (kb) in Fayoumi/Leghorn vs. RJF and Fayoumi vs. Leghornized reference. Shape of the graph shows the amount of variability still present on chromosome 16 despite high levels of homozygosity for each population vs. the reference genome and for the Fayoumi vs. Leghorn alternate reference. The MHC regions are highlighted to show differences in variation possibly related to the difference in pathogen resistance between the two populations. The y-axis represents variants, x-axis position, and the dashed lines show peak heights for the first 250 Kb of the chromosome. Fayoumi vs. leghorn alternate reference is based on SNV comparison only

To facilitate between-line analysis of variants and reduce bias related to choice of the reference sequence, the nucleotides in the RJF reference genome were replaced with the discovered Leghorn variants to create an alternate genome assembly. The Fayoumi was then realigned to this new reference for variant analysis. When compared for SNVs against the Leghorn, the Fayoumi displayed a total of 3,792,327 differences. Of these differences, 2,697 had high and 25,095 had moderate impacts, numbers higher than the 1,130 high and 15,468 moderate impact variants called in Fayoumi against the RJF sequence. In this comparison to the Leghorn, the Fayoumi had less 5’UTR, and downstream annotated variants than were called against the RJF sequence (Table 9). To further elucidate differences between the lines, a GO analysis was done on the genes that contained exon variants that were fixed in the Fayoumi. Many of the moderate impact variants that were called for exon effects (*N* = 300) were located in micro-RNAs and small nuclear RNAs e.g. gga-mir-6676 and gga-mir-6616, which are related to chicken gastrulation and embryogenesis [34, 35] (Additional file 2: Table S2). The limited overlap between reproduction and immune related functions indicate a breed difference between Fayoumi and Leghorn in production/reproduction genotypes. A comparison of variant frequency across each chromosome of the Fayoumi genome when aligned against the Leghornized reference showed large areas of homology between the two lines for most chromosomes. On chromosome 16 (length ~540 Kb), which harbors the chicken MHC complex, the two breeds show differences in the total number of changes across the chromosome. Chromosome 16 was the third most variable chromosome in the Leghorn population with a variant every 180 base pairs and the fifth most variable in the Fayoumi population with a variant every 189 base pairs. The amount of variance within each population for chromosome 16 was less than 1 % (0.54 % on average), despite showing differences in the number of variants within the MHC-B region (0-250 Kb). The breed variation is represented by 1,936 SNVs that differ between Fayoumi and Leghorn, based upon alignment (Fig. 1). The differences in SNV counts may be responsible for the disparity between the immune response of the Fayoumi and Leghorn or represent a higher standing genetic variation, as seen in the difference in total number of heterozygous variants, which is higher in the Fayoumi.

## Conclusions

The study elucidated variants indicative of a genetic foundation for characteristic breed phenotypes (Fayoumi = immune traits, Leghorn = reproductive traits). The low levels of within-line variation were consistent with the lines’ extreme levels of inbreeding and the high between-line variation was concordant with the lines’ diverse backgrounds. The Leghorn had more fixed variants and the Fayoumi more heterozygous variants, compared to the RJF reference. Greater fixation in the Leghorn line may be a result of stringent historical selection for a limited number of traits in this breed, where as greater genomic heterozygosity may be an advantage for disease-resistance traits in the Fayoumi. For both lines, most variants were in intergenic and intronic regions, limiting their impact on the survivability of the populations. The major genetic differences between breeds by *F*_*ST*_ and subsequent GSEA were consistent with the overarching phenotype ascribed to the lines; thus, the study’s data aligned well with the breed characteristics and supported a connection of breed-predominant phenotypes with the genomic variation in the lines. Additionally, the GSEA results from the strict filtered data reinforced the correspondence between the breed-predominant phenotypes and the biological processes, functions, and genes that were elucidated by original GSEA and GO analysis. The table generated by the stricter parameters does, however, give more specific terms related to immune functions and the structural components of eggs related to the breed phenotypes. The study lays the foundation to elucidate and verify differences in function caused by the unique variants found within the populations representing the two breeds.

## Methods

### Animals

The chickens were produced and maintained in the Iowa State University poultry genetics program. The Fayoumi and Leghorn breeds broadly represent a divergent history of either natural selection for disease resistance or artificial selection for reproduction, respectively [30, 36]. The Fayoumi line was established from birds imported to the USA in 1954 because of reported genetic resistance to viral disease. The Leghorn line was established from commercial Leghorn layer lines sourced in the U.S.A. in 1954. The birds characterized in this study are extremely inbred, having been sib mated for over 70 generations, since 1954. The inbreeding process is assumed to have, on average, moved toward fixation the alleles that were in highest frequency in the founder individuals of these lines. Since inception of inbreeding, the only phenotypes under selection were those required to propagate the lines (general survivability and reproduction), and this selection occurred equally in both lines.

### DNA extraction and resequencing

The DNA from 16 birds per line were pooled by line, in equal quantities, and used for resequencing. DNA was isolated from blood using an in house DNA isolation procedure. Quality and concentration were determined through NanoDrop testing. DNA was sequenced using DNA Landmarks via the Hiseq 2000 using TruSeq V3 chemistry.

### Alignment and mapping of sequence reads

The Burrows-Wheeler Aligner was used to align sequence reads to the Galgal4 reference genome using the default settings for gap extensions, gap and mismatch penalties [37]. The Sampe setting was used for SAM file generation. SAM files were then converted to BAM files and sorted using Samtools [38, 39]. The files for both breeds were corrected for any errors that may have resulted from file conversion using Picard [39] prior to variant discovery. Assembly coverage was calculated using Samtools for alignment of the Fayoumi and Leghorn samples to the Red Jungle Fowl reference (Table 2). The DepthofCoverage tool in GATK [40] was used to calculate the sequence coverage (Table 2).

### Variant discovery

SNVs and indels were called using the Genome Analysis Tool Kit Unified Genotyper (GATK-UG) tool. The GATK-UG has the ability to call variant sites within pooled samples and thereby provide an estimate of allele counts and frequency within a population [40, 41]. The GATK-UG was run using parameter arguments that allowed use of the GLM method for discovery of both SNVs and indels that had a minimum phred-scaled confidence threshold of 50 to call variants. Down sampling was turned off so as to not bias the variant discovery, and the ploidy option were used to account for the 16 individuals in each pool to get the correct allele frequencies.

### Alternate reference genome creation

To facilitate a direct comparison of the variants contained within the Fayoumi and Leghorn populations, an *in silico* reference genome based on one of the experimental populations was created with the GATK FastaAlternateReferenceMaker option. The Leghorn vcf file was used as a variant file to replace the RJF reference alleles with Leghorn variants. Leghorn vcf was chosen to create the alternate reference because it is the breed most used for commercial white-egg layers and could be useful for other comparisons of lines of commercial interest. This tool can only lift over consensus Leghorn SNV positions (not indels) to the reference assembly. Because of this limitation, all downstream analyses of the Fayoumi vs. Leghorn data were based only on SNVs.

### Functional annotation (SnpEff and SNPSift)

Gene annotation and prediction of the functional consequences of variants was done with SnpEff [42]. Each variant was annotated by type (none, chromosome, cds, intergenic, intergenic_conserved, upstream, utr_5_prime, utr_5_deleted, start_gained, splice_site_acceptor, splice_site_donor, intragenic, start_lost, intron, utr_3_prime, utr_3_deleted, downstream, etc.) and region (exon, intron, intergenic, splice_site_acceptor, splice_site_donor) and the functional annotation (nonsynonymous, synonymous, stop_codon gain_loss, and amino_acid_change) based on the Galgal4 reference genome. These files were then filtered for known dbSNP variants from Ensembl [43] and a quality score ≥ 50, depth of coverage ≥ 2, and minor allele frequency (MAF) of 0.3, with all other parameters set at default. Median depth of coverage was 43 for Fayoumi and 38 for Leghorn samples. Variants were also examined and tagged for loss of function (lof) mutations under the same parameters as the aforementioned variants. After validation, a stricter set of filtering parameters was used to address and reduce possible genotyping errors and duplications. The stricter parameters were used to generate gene lists to explore possible over-enrichment of biological processes and functions based upon the variation that exists within each population. The parameters used for the strict filtering of the variants included: MAF ≥ 0.25 also any allele frequency (AF) = 0.50 was removed to address duplications. The depth of coverage (DP) was based on a range from 43–73 to account for duplications seen during SNV validation. The range for DP is based 2sd (1sd = ~15) of the median DP. Quality per base was based on median quality /# of birds in pool (1500/16). Lastly, only the moderate effects were used to allow for comparison to the previous list (exploratory filters).

### Fixation index analysis (*F*_*ST*_)

The fixation analysis was performed using PoPoolation2 [28] to examine genomic differentiation between the populations. The data was prepared by first mapping the sequencing data to the RJF reference genome then using Samtools [44, 45] ambiguous reads were removed. The mpileup function was then used to generate sync files containing the allele frequencies for each population at each locus and for each gene within the genome. PoPoolation2 [31] was then used calculate the allele frequency differences based on a pairwise comparison of the populations for each gene in the Galgal4.72.gtf file by sliding window analysis. The Karlsson *F*_*ST*_ method [2] was used with the following parameters --min-count 3, −-min-coverage 3, −-max-coverage 2 %, −-window-size 1, −-step-size 1, and --pool-size 16:16. Downstream GSEA analysis was conducted on genes showing an *F*_*ST*_ value of 1.0 to represent genes showing possible differences in function between the populations.

### Gene set enrichment analysis (GSEA) and gene ontology (GO) analysis

For the within population data analysis, gene set enrichment analysis (GSEA) and GO analysis was performed using DAVID [44] and (GO)TermFinder [45]. Visualization of enrichment results was done using REViGO [29]. The additional analysis of the *F*_*ST*_ data was carried out in REViGO, which reduces the list of terms based on uniqueness and dispensability. Uniqueness is a measure of whether the term is an outlier when compared semantically to the list of generated GO terms. In addition, the program also reduces the functional redundancies [46] by filtering semantically similar terms to allow for a single GO term to represent a cluster. The original gene list used in DAVID [44] was re-analyzed using both DAVID [44] and (GO)TermFinder [45] based on gene list created from the strict filters applied to the within line variant data for each population. All software was run using the default parameters for both the exploratory and strict filtered gene lists. Only the annotations for chicken were used and based on background lists for ~17,000 annotated genes for the chicken reference genome. The experimental gene lists generated for analysis consisted of Fayoumi (unique, fixed/segregating variant gene list) = 7,688 and Leghorn (unique, fixed/segregating variant gene list) = 10,807. Genes containing multiple variants were only supplied once to the programs. This list included both miRNA and snRNA containing variants. Only the genes showing unique variants (fixed or segregating) of moderate impact were supplied as gene list to the analysis software. From the *F*_*ST*_ analysis, a list of 9,573 genes was used for the *F*_*ST*_ GSEA analysis.

## Additional files

Additional file 1: Table S1. Strict filtered gene list for overrepresented gene ontology terms for moderate impact^§^, line-specific variants in Fayoumi and Leghorn lines. Table shows the statistically significant (*FDR ≤ 0.05*)^§^ GO terms related to the historical breed phenotypes for each of the inbred populations. (DOCX 18 kb)
Additional file 2: Table S2. Fayoumi vs. Leghorn alternate reference genes with exonic SNVs. Genes in list are from exploratory filter and number and state of variants represents data for SNV changes only. Variants are either fixed or segregating within the Fayoumi population. (DOCX 111 kb)

## Abbreviations

AF: allele frequency
DP: depth of coverage
FST: fixation index test
MAF: minor allele frequency
NGS: next generation sequencing
SNV: single nucleotide variant
