# Supplementary material for: Single nucleotide variant discovery of highly inbred Leghorn and Fayoumi chicken breeds using pooled whole genome resequencing data reveals insights into phenotype differences
Source: BMC Genomics. 2016 Oct 19;17:812. doi: 10.1186/s12864-016-3147-7 (PMC5070165; doi:10.1186/s12864-016-3147-7)
Supplement: Additional file 1: Table S1. — Strict filtered gene list for overrepresented gene ontology terms for moderate impact§, line-specific variants in Fayoumi and Leghorn lines. Table shows the statistically significant (FDR ≤ 0.05)§ GO terms related to the historical breed phenotypes for each of the inbred populations. (DOCX 18 kb) [file 12864_2016_3147_MOESM1_ESM.docx]

| Population | GO Term | Count | Relation to breed phenotype |
| --- | --- | --- | --- |
| Fayoumi (Strict filter) | FN3 | 37 | Cytokine signaling, wound healing |
|  | Fibronectin, type III-like fold | 38 | Cytokine signaling, wound healing |
|  | Natural killer cell | 4 | Immune system, viral pathogens |
|  | Cytokine-cytokine receptor interaction | 149 | Immune system, cytokine signaling |
| Leghorn (strict filter) | Homophilic cell adhesion | 33 | Eggshell matrix, cadherin |
|  | Biological adhesion | 59 | Eggshell matrix |
|  | CA | 19 | Eggshell matrix, Ca2+-mediated cell-cell adhesion |
|  | Cadherin | 19 | Eggshell matrix, Ca2+-mediated cell-cell adhesion |
|  | Calcium ion binding | 76 | Eggshell matrix |
|  | Glycerophospholipid metabolic process | 28 | Egg lipid matrix |
|  | Extracellular matrix | 41 | Eggshell matrix |
|  | Oxoacid metabolic process | 72 | Proteoglycans |

**Additional file 1: Table S1.** Strict filtered gene list for overrepresented gene ontology terms for moderate impact^§^, line-specific variants in Fayoumi and Leghorn lines.

Table shows the statistically significant (*FDR ≤ 0.05*)^§^ GO terms related to the historical breed phenotypes for each of the inbred populations.
