# Supplementary material for: Single nucleotide variant discovery of highly inbred Leghorn and Fayoumi chicken breeds using pooled whole genome resequencing data reveals insights into phenotype differences
Source: BMC Genomics. 2016 Oct 19;17:812. doi: 10.1186/s12864-016-3147-7 (PMC5070165; doi:10.1186/s12864-016-3147-7)
Supplement: Additional file 2: Table S2. — Fayoumi vs. Leghorn alternate reference genes with exonic SNVs. Genes in list are from exploratory filter and number and state of variants represents data for SNV changes only. Variants are either fixed or segregating within the Fayoumi population. (DOCX 111 kb) [file 12864_2016_3147_MOESM2_ESM.docx]

Additional file 2: Table S2. Fayoumi vs. Leghorn alternate reference genes with exonic SNVs

| GeneID | # of exonic variants | State | GeneID | # of exonic variants | State |
| --- | --- | --- | --- | --- | --- |
| 5S_rRNA | 5 | Fixed | ENSGALG00000003330 | 2 | Segregating |
| 7SK | 1 | Fixed | ENSGALG00000026343 | 1 | Segregating |
| ABCA7 | 2 | Fixed | ENSGALG00000026508 | 2 | Segregating |
| AIRE | 2 | Fixed | ENSGALG00000027373 | 1 | Segregating |
| ASH1L | 2 | Fixed | ENSGALG00000027773 | 1 | Segregating |
| C19H17ORF85 | 1 | Fixed | gga-mir-1670 | 2 | Segregating |
| CAND2 | 2 | Fixed | gga-mir-6559 | 1 | Segregating |
| EGOT | 1 | Fixed | gga-mir-6610 | 1 | Segregating |
| ENSGALG00000000188 | 5 | Fixed | Metazoa_SRP | 20 | Segregating |
| ENSGALG00000003330 | 8 | Fixed | NIPA1 | 4 | Segregating |
| ENSGALG00000003670 | 1 | Fixed | SNORA18 | 4 | Segregating |
| ENSGALG00000015400 | 3 | Fixed | uc_338 | 3 | Segregating |
| ENSGALG00000018702 | 2 | Fixed |  |  |  |
| ENSGALG00000018733 | 3 | Fixed |  |  |  |
| ENSGALG00000020517 | 1 | Fixed |  |  |  |
| ENSGALG00000021180 | 1 | Fixed |  |  |  |
| ENSGALG00000025316 | 1 | Fixed |  |  |  |
| ENSGALG00000025414 | 2 | Fixed |  |  |  |
| ENSGALG00000025733 | 1 | Fixed |  |  |  |
| ENSGALG00000025962 | 2 | Fixed |  |  |  |
| ENSGALG00000026432 | 1 | Fixed |  |  |  |
| ENSGALG00000026632 | 1 | Fixed |  |  |  |
| ENSGALG00000027016 | 1 | Fixed |  |  |  |
| ENSGALG00000027055 | 3 | Fixed |  |  |  |
| ENSGALG00000027223 | 1 | Fixed |  |  |  |
| ENSGALG00000027373 | 10 | Fixed |  |  |  |
| ENSGALG00000027422 | 1 | Fixed |  |  |  |
| ENSGALG00000027568 | 1 | Fixed |  |  |  |
| ENSGALG00000027683 | 3 | Fixed |  |  |  |
| ENSGALG00000027832 | 1 | Fixed |  |  |  |
| ENSGALG00000027979 | 2 | Fixed |  |  |  |
| ENSGALG00000028261 | 1 | Fixed |  |  |  |
| ENSGALG00000028319 | 1 | Fixed |  |  |  |
| ENSGALG00000028381 | 4 | Fixed |  |  |  |
| ENSGALG00000028382 | 1 | Fixed |  |  |  |
| ENSGALG00000028490 | 1 | Fixed |  |  |  |
| ENSGALG00000028511 | 2 | Fixed |  |  |  |
| ENSGALG00000028596 | 1 | Fixed |  |  |  |
| FAM60A | 4 | Fixed |  |  |  |
| gga-let-7a-1 | 1 | Fixed |  |  |  |
| gga-let-7f | 1 | Fixed |  |  |  |
| gga-mir-100 | 2 | Fixed |  |  |  |
| gga-mir-1354 | 1 | Fixed |  |  |  |
| gga-mir-135a-3 | 1 | Fixed |  |  |  |
| gga-mir-1462 | 2 | Fixed |  |  |  |
| gga-mir-1550 | 1 | Fixed |  |  |  |
| gga-mir-1559 | 1 | Fixed |  |  |  |
| gga-mir-1565 | 1 | Fixed |  |  |  |
| gga-mir-1567 | 1 | Fixed |  |  |  |
| gga-mir-1568 | 1 | Fixed |  |  |  |
| gga-mir-1579 | 1 | Fixed |  |  |  |
| gga-mir-1587 | 1 | Fixed |  |  |  |
| gga-mir-1596 | 1 | Fixed |  |  |  |
| gga-mir-1601 | 1 | Fixed |  |  |  |
| gga-mir-1604 | 2 | Fixed |  |  |  |
| gga-mir-1608 | 5 | Fixed |  |  |  |
| gga-mir-1616 | 1 | Fixed |  |  |  |
| gga-mir-1688 | 1 | Fixed |  |  |  |
| gga-mir-1704 | 2 | Fixed |  |  |  |
| gga-mir-1727 | 2 | Fixed |  |  |  |
| gga-mir-1728 | 1 | Fixed |  |  |  |
| gga-mir-1736 | 4 | Fixed |  |  |  |
| gga-mir-1738 | 2 | Fixed |  |  |  |
| gga-mir-1757 | 1 | Fixed |  |  |  |
| gga-mir-1778 | 2 | Fixed |  |  |  |
| gga-mir-1789 | 1 | Fixed |  |  |  |
| gga-mir-1794 | 1 | Fixed |  |  |  |
| gga-mir-1806 | 1 | Fixed |  |  |  |
| gga-mir-1807 | 2 | Fixed |  |  |  |
| gga-mir-18b | 1 | Fixed |  |  |  |
| gga-mir-194 | 2 | Fixed |  |  |  |
| gga-mir-199-1 | 1 | Fixed |  |  |  |
| gga-mir-20b | 1 | Fixed |  |  |  |
| gga-mir-216a | 3 | Fixed |  |  |  |
| gga-mir-222a | 1 | Fixed |  |  |  |
| gga-mir-3523 | 1 | Fixed |  |  |  |
| gga-mir-3536 | 1 | Fixed |  |  |  |
| gga-mir-455 | 1 | Fixed |  |  |  |
| gga-mir-458a | 1 | Fixed |  |  |  |
| gga-mir-460a | 1 | Fixed |  |  |  |
| gga-mir-460b | 1 | Fixed |  |  |  |
| gga-mir-6542 | 2 | Fixed |  |  |  |
| gga-mir-6579-2 | 2 | Fixed |  |  |  |
| gga-mir-6583 | 1 | Fixed |  |  |  |
| gga-mir-6600 | 2 | Fixed |  |  |  |
| gga-mir-6602 | 1 | Fixed |  |  |  |
| gga-mir-6607 | 2 | Fixed |  |  |  |
| gga-mir-6610 | 2 | Fixed |  |  |  |
| gga-mir-6616 | 1 | Fixed |  |  |  |
| gga-mir-6631 | 1 | Fixed |  |  |  |
| gga-mir-6641 | 2 | Fixed |  |  |  |
| gga-mir-6666 | 2 | Fixed |  |  |  |
| gga-mir-6671 | 3 | Fixed |  |  |  |
| gga-mir-6674 | 3 | Fixed |  |  |  |
| gga-mir-6675 | 1 | Fixed |  |  |  |
| gga-mir-6676 | 2 | Fixed |  |  |  |
| gga-mir-6679 | 1 | Fixed |  |  |  |
| gga-mir-6692 | 1 | Fixed |  |  |  |
| gga-mir-6693 | 5 | Fixed |  |  |  |
| gga-mir-6703 | 1 | Fixed |  |  |  |
| gga-mir-6713 | 1 | Fixed |  |  |  |
| gga-mir-99a | 1 | Fixed |  |  |  |
| IBR/F | 1 | Fixed |  |  |  |
| MIEN1 | 2 | Fixed |  |  |  |
| NUP93 | 1 | Fixed |  |  |  |
| RAB7L1 | 1 | Fixed |  |  |  |
| RPS3 | 1 | Fixed |  |  |  |
| SCARNA11 | 2 | Fixed |  |  |  |
| SCARNA6 | 3 | Fixed |  |  |  |
| SNORA18 | 2 | Fixed |  |  |  |
| SNORA21 | 3 | Fixed |  |  |  |
| SNORA23 | 1 | Fixed |  |  |  |
| SNORA3 | 1 | Fixed |  |  |  |
| SNORA36 | 4 | Fixed |  |  |  |
| SNORA5 | 6 | Fixed |  |  |  |
| SNORA53 | 1 | Fixed |  |  |  |
| SNORA65 | 3 | Fixed |  |  |  |
| SNORA74 | 2 | Fixed |  |  |  |
| SNORA8 | 3 | Fixed |  |  |  |
| SNORA9 | 2 | Fixed |  |  |  |
| SNORD102 | 1 | Fixed |  |  |  |
| SNORD15 | 2 | Fixed |  |  |  |
| SNORD17 | 1 | Fixed |  |  |  |
| SNORD37 | 1 | Fixed |  |  |  |
| SNORD53_SNORD92 | 2 | Fixed |  |  |  |
| SNORD97 | 3 | Fixed |  |  |  |
| snoU83B | 1 | Fixed |  |  |  |
| snoU85 | 2 | Fixed |  |  |  |
| TMEM110 | 3 | Fixed |  |  |  |
| U1 | 2 | Fixed |  |  |  |
| U6 | 2 | Fixed |  |  |  |
| U6atac | 3 | Fixed |  |  |  |
| uc_338 | 11 | Fixed |  |  |  |
| UNC45B | 1 | Fixed |  |  |  |
| UPF3B | 1 | Fixed |  |  |  |

Genes in list are from exploratory filter and number and state of variants represents data for SNV changes only. Variants are either fixed or segregating within the Fayoumi population.
